# Stretchable, Patterned Carbon Nanotube Array Enhanced by Ti_3_C_2_T_x_/Graphene for Electromagnetic Interference Shielding

**DOI:** 10.3390/nano15050391

**Published:** 2025-03-03

**Authors:** Baohua Li, Xuebin Liu, Jiyong Feng, Yunfan Wang, Junhua Huang, Zhengwei Fu, Zhiping Zeng, Jianghui Zheng, Xuchun Gui

**Affiliations:** 1 State Key Laboratory of Optoelectronic Materials and Technologies, School of Electronics and Information Technology, Sun Yat-sen University, Guangzhou 510275, China; 2 Beijing Zhenxing Institute of Metrology and Measurement, Beijing 100074, China; 3 School of Materials Science and Engineering, Sun Yat-sen University, Guangzhou 510275, China

**Keywords:** carbon nanotube array, Ti_3_C_2_T_x_, film, electromagnetic interference shielding

## Abstract

Stretchability and flexibility are essential characteristics for high-performance electromagnetic interference (EMI) shielding materials in wearable and smart devices. However, achieving these mechanical properties while also maintaining high EMI shielding effectiveness (SE) for shielding materials remains a significant challenge. Here, a stretchable patterned carbon nanotube (CNT) array composite film, reinforced with two-dimensional (2D) nanomaterials (Ti_3_C_2_T_x_ and graphene), is fabricated using a straightforward scraping method. The resulting CNT array/Ti_3_C_2_T_x_/graphene composite films possess a periodic grid structure. Specifically, the composite film with a regular hexagonal pattern demonstrates an EMI SE of 36.5 dB in the X-band at a thickness of 350 μm. Additionally, the composite film exhibits excellent stretchability, flexibility, and stability. After undergoing 10,000 stretching cycles, the EMI SE remains stable. Simulation results further indicate that surface reflection is the primary EMI shielding mechanism. This simple scraping method offers a promising approach for developing stretchable and high-performance EMI shielding films, making them well suited for application in flexible devices.

## 1. Introduction

The booming development of mobile communication devices and flexible electronics has greatly improved our daily lives. However, the resulting electromagnetic radiation pollution can lead to the failure of electronic devices, which threatens their normal functionality and can even pose serious risks to human health [1,2,3,4,5,6]. Consequently, the demand for efficient electromagnetic interference (EMI) shielding materials is pressing and imperative [7,8,9,10]. Unlike traditional metal-based materials, the next generation of EMI shielding materials for flexible electronics is required to possess characteristics such as high efficiency, stretchability, flexibility, and being lightweight. Carbon-based nanomaterials, including carbon nanotubes (CNTs) [11,12,13], carbon nanofiber [14,15], and graphene [16,17], are considered promising EMI shielding materials due to their excellent electrical properties. For example, CNT sponge/epoxy resin composite material with a thickness of 2.0 mm exhibits an EMI shielding effectiveness (SE) of 33 dB in the X-band [11]. The graphene foam shows an EMI SE of 30 dB at a thickness of 1.0 mm [16]. However, in the above-mentioned carbon-based porous materials, the shielding performance highly depends on the thickness of the sample [12,17]. Generally, carbon-based materials require a relatively large thickness to achieve remarkable electromagnetic shielding effectiveness [18,19,20,21]. Therefore, developing flexible, high-performance EMI shielding materials that maintain limited thickness remains a significant challenge.

Recently, CNTs have gained widespread attention, due to their excellent processability, extraordinary mechanical strength, and outstanding electrical conductivity. Nevertheless, the EMI performance of CNT film is significantly dependent on its thickness [22,23,24]. One effective approach to enhance the EMI performance of CNTs is to improve the reflection capability of shielding materials against electromagnetic waves. Numerous efforts have been made to increase the conductivity, as it directly determines the reflection loss of the samples. Ti_3_C_2_T_x_, as a typical MXene material, is a new class of transition metal carbide materials with a unique two-dimensional flake-like structure, and excellent metal-like electrical conductivity. These characteristics enhance the possibility of scattering and absorption of electromagnetic waves that enter the materials, resulting in highly efficient electromagnetic shielding [2,25,26,27,28,29,30,31,32,33,34]. For instance, Ti_3_C_2_T_x_ films can achieve high EMI SE at relatively low thicknesses (45 μm, 92 dB@8.2 GHz) [2]. However, the weak interlayer interaction force between Ti_3_C_2_T_x_ nanosheets results in limited flexibility and stretchability in Ti_3_C_2_T_x_-based films [33]. By combining CNT with Ti_3_C_2_T_x_, it is possible to achieve high-performance EMI shielding while maintaining flexibility and stretchability. For example, the flexible CNT/Ti_3_C_2_T_x_/cellulose nanofiber hybrid membrane exhibits a high conductivity of 2506.6 S/m and an SE of 38.4 dB in the X-band at a thickness of 38 μm [35]. Additionally, an aligned CNT array/Ti_3_C_2_T_x_ composite film obtained by the chemical crosslinking method achieves an EMI SE of 70 dB at a thickness of 28 μm, with a specific SE reaching 122,368 dB cm^2^/g [36]. Nonetheless, challenges such as uneven stacking of Ti_3_C_2_T_x_ during the composite process and the overly complex layer-by-layer assembly processes can negatively affect the mechanical properties [2,37,38]. In addition, the complete transfer of the CNT array is also a crucial issue. Recently, complete transfer of vertically aligned CNT arrays onto the surface of prepreg has been achieved through the capillary impregnation method [39]. Therefore, it is necessary to develop novel methods for compositing CNT arrays with Ti_3_C_2_T_x_ to achieve excellent EMI shielding with outstanding flexibility.

Herein, a composite approach has been designed to fabricate flexible Ti_3_C_2_T_x_/graphene-enhanced CNT array films with high EMI SE. The CNT arrays, which feature various patterns (hexagon, diamond, and triangle), are first synthesized using chemical vapor deposition (CVD). Then, the Ti_3_C_2_T_x_/graphene mixed slurry is uniformly filled into the “cells” of the patterned CNT array by a scraping method. Ti_3_C_2_T_x_ is primarily aimed at increasing the electrical conductivity of the film, while the graphene is used to support Ti_3_C_2_T_x_ and enable their co-compounding into the CNT array. The resulting composite film with a regular hexagonal pattern achieves an EMI SE of 36.5 dB at a thickness of 350 μm. The composite films also exhibit favorable stretchability and stability, while the EMI SE can remain stable after fatigue stretching. Specifically, the EMI SE remains stable even after 10,000 stretching cycles at 40% tensile strain. The shielding mechanism of the composite film has been confirmed through simulation using CST software (CST Studio Suite 2022, v1.0.0). The results indicate that the reflection is the primary shielding mechanism.

## 2. Experimental Section

### 2.1. Preparation of Patterned CNT Array

The silicon wafers were cleaned sequentially with acetone, isopropyl alcohol, and deionized water first. Then, a Ni/Au layer was deposited onto the silicon substrate by electron beam evaporation. Various Ni/Au patterns were fabricated by utilizing a mask plate with different designs. Subsequently, a patterned CNT array was synthesized on these substrates through the CVD method, as reported in earlier work [40]. The thicknesses of the CNT array could be easily controlled by adjusting the growth time.

### 2.2. Preparation of Ti_3_C_2_T_x_/Graphene Mixed Slurry

The graphene was sourced from XFNano Materials Tech Co (Nanjing, China). Ti_3_C_2_T_x_ nanosheets were prepared by etching MAX phase precursor. The preparation process is as follows: First, 2 g LiF was added to 30 mL 9 M HCl. The mixture was stirred at 35 °C for 30 min and then 2 g of Ti_3_AlC_2_ was slowly added into the mixed solution. The Ti_3_C_2_T_x_ precipitate was then repeatedly washed with deionized water until the pH was 7. During each washing process, the Ti_3_C_2_T_x_ precipitate was separated by centrifugation at 5000 rpm for 5 min. Then, the Ti_3_C_2_T_x_ powder was re-dispersed to deionized water and sonicated for 2 h in an ice bath. The resulting dispersion was centrifuged at 8000 rpm for 5 min and the supernatant was freeze-dried for 72 h. Finally, delaminated Ti_3_C_2_T_x_ nanosheet powder was obtained after freeze-drying, as reported in our early work [19]. After that, 0.2 g Ti_3_C_2_T_x_ nanosheets was mixed with 17.3 mL deionized water, and the mixture was subjected to ultrasound for 15 min to achieve a uniformly mixed Ti_3_C_2_T_x_ aqueous solution. Then, 2.5 g graphene slurry (solid content 8%) was thoroughly stirred for 2 h and added into the Ti_3_C_2_T_x_ solution. Finally, a 10 mg/mL Ti_3_C_2_T_x_/graphene mixed slurry was obtained by stirring the mixture evenly for an additional 2 h.

### 2.3. Preparation of CNT Array/Ti_3_C_2_T_x_/Graphene/PDMS Composite Films

The CNT array was initially treated with oxygen plasma for 5 min. Then, the Ti_3_C_2_T_x_/graphene mixed slurry was uniformly applied to the patterned CNT array by scraping. The scraping process was repeated 3 times, after which the array was allowed to dry at room temperature. After that, polydimethylsiloxane (PDMS) was slowly poured into the dried CNT array/Ti_3_C_2_T_x_/graphene composite material and degassed under vacuum for uniform coating. The composite film was cured at 65 °C for 3 h in an oven. Finally, the CNT array/Ti_3_C_2_T_x_/graphene/PDMS composite film was obtained by transferring and carefully peeling it off the silicon substrate.

### 2.4. Characterization

The morphology of the composite films was characterized using scanning electron microscopy (Hitachi, S-4800, Japan). Sheet resistances were measured with a 4-point probe resistivity measurement system (4 probes tech, China). Raman spectroscopy (HORIBA, LabRAMHR, Japan) and X-ray photoelectron spectroscopy (XPS; Thermo Scientific, ESCALAB 250Xi, Massachusetts, USA) were employed to analyze the chemical bonds and states of the composite films. For the Raman testing, a laser with a wavelength of 633 nm and power of 17.5 mW was used. The mechanical endurance and stability were assessed using a universal testing machine (Instron, 5943, Boston, USA).

### 2.5. EMI Shielding Effectiveness

The EMI SE was measured using a vector network analyzer (N5232, VNA, Keysight, Santa Rosa, USA) based on the waveguide method within an 8.2–12.4 GHz region (X-band). The EMI SE of different samples was also tested in the full spectrum of 8.2–40 GHz. According to the possible mechanism of the composite film, the parameters *A*, *R*, and *T* represented the absorption, reflection, and transmission ratios, respectively, which were obtained by calculating the *S* parameters. Once the above parameters were determined, the EMI SE could also be calculated using the following formulas [41]:

(1) $$\begin{array}{c} R={\left|S11\right|}^{2}={\left|S22\right|}^{2} \end{array}$$

(2) $$\begin{array}{c} T={\left|S21\right|}^{2}={\left|S12\right|}^{2} \end{array}$$

(3) $$\begin{array}{c} A+R+T=1 \end{array}$$

(4) $$\begin{array}{c} {SE}_{T}\left(dB\right)=-10logT \end{array}$$

(5) $$\begin{array}{c} {SE}_{R}\left(dB\right)=-10log\left(1-R\right) \end{array}$$

(6) $$\begin{array}{c} {SE}_{A}\left(dB\right)={SE}_{T}-{SE}_{R} \end{array}$$

where the *SE_T_*, *SE_R_*, and *SE_A_* represent the total EMI SE, the reflective EMI SE, and the absorptive EMI SE, respectively. In addition, *Sij* indicates that electromagnetic waves are transmitted from port *i* to port *j* and the |*Sij*|^2^ represents the power of the electromagnetic waves transferred from port *i* to port *j*.

### 2.6. Simulation

The electromagnetic simulation software CST (CST Studio Suite 2022, v1.0.0) was used for the simulations. The material parameters were defined as follows: The background material was configured as air first, with a dielectric constant of 1.0. Then, in the case of PDMS, the dielectric constant was established at 2.7, with a magnetic loss tangent of 0.03. The dielectric constant for the CNT array was set at 2.2, and its electrical conductivity was set at 180 S/m. Since Ti_3_C_2_T_x_ and graphene were used as a mixed slurry and the scraping and coating operations during actual processes were conducted three times, the electrical conductivity was defined as 3000 S/m [42]. After setting these parameters, different pattern structures could be designed, allowing for the corresponding EMI SE results to be obtained.

## 3. Results and Discussion

**Figure 1:**
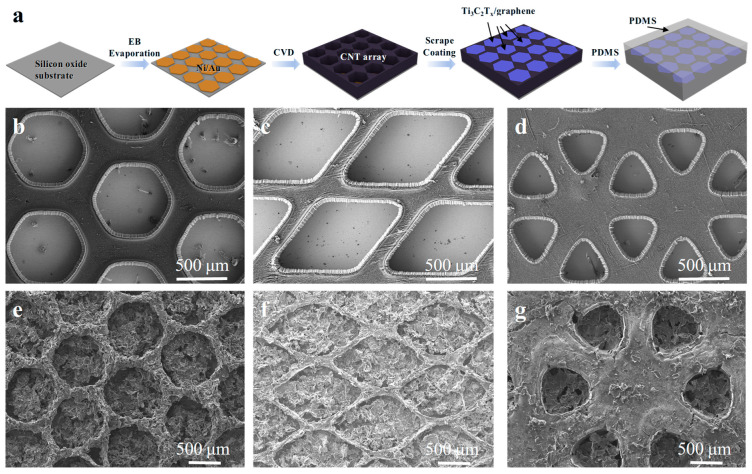
Fabrication and morphology of the composite films. (**a**) Schematic diagrams of the fabrication process for composite films. Each material is clearly represented in the figure with a different color. (**b**–**d**) SEM images of CNT array films with the periodic hexagonal, diamond, and triangle patterns, respectively. (**e**–**g**) SEM images of composite films (after compositing with Ti_3_C_2_T_x_/graphene) with the periodic hexagonal, diamond, and triangle patterns, respectively.

The fabrication process for the CNT array/Ti_3_C_2_T_x_/graphene/PDMS composite films is demonstrated in Figure 1a. Firstly, a Ni/Au (25/70 nm) pattern with a periodic shape, including hexagons, diamonds, and triangles, is deposited onto a silicon sheet using electron beam evaporation. These periodic shape designs are relatively simple, have good stability, and are beneficial for maintaining the overall structural stability of the composite film after scrape coating with Ti_3_C_2_T_x_/graphene. Next, this silicon sheet serves as the substrate for the CNT array growth. Due to the inhibiting effect of Au on CNT growth, the CNT array will not grow in areas that have been coated with Ni/Au, but it will grow in the uncoated areas, resulting in a continuous grid structure [40]. The thickness of the CNT array can be controlled by adjusting the growth time (Figure S1). Thirdly, the Ti_3_C_2_T_x_/graphene mixture is filled into the “cells” of the CNT array grid by a scraping method. Finally, PDMS is poured over the film, allowing it to penetrate the interior of the CNT film under capillary force. Once the sample is completely cured, it can be peeled off from the substrate to yield a flexible composite film. Figure 1b–d present SEM images of CNT arrays with the patterns of regular hexagons, diamonds, and triangles, respectively. In all samples, the CNT array forms a continuous film with periodic small “cells” that did not grow the CNT array. The SEM images reveal that the CNTs are organized in an orderly and parallel arrangement within the films (Figure S1). The diameter of the CNTs is approximately several tens of nanometers, with noticeable gaps between the tubes, facilitating subsequent composite formation with PDMS. After scraping Ti_3_C_2_T_x_/graphene slurry in the CNT array, the periodic structure of the array grid remains intact, with the Ti_3_C_2_T_x_/graphene uniformly filling the “cells”. Additionally, a small amount of nanosheets are observed on the surface of the array (Figure 1e–g and Figure S2). When PDMS is incorporated into the CNT array, the resulting composite can be smoothly peeled from the substrate.

**Figure 2:**
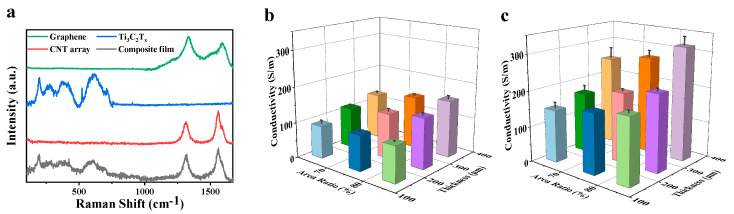
Characterization and electrical conductivity of the films. (**a**) Raman spectra of graphene, Ti_3_C_2_T_x_, the initial CNT array, and the composite film. (**b**) The conductivity of the initial CNT array with hexagonal pattern at different thicknesses and area ratios. (**c**) The conductivity of the composite films with hexagonal patterns at different thicknesses and area ratios.

Figure 2a presents the Raman spectra of graphene, Ti_3_C_2_T_x_, the initial CNT array, and the composite film. Both CNT array and the graphene sample exhibit distinct peaks at approximately 1350 cm^−1^ and 1580 cm^−1^, which correspond to the characteristic D peak and G peak of carbon, respectively. In addition, Ti_3_C_2_T_x_ displays characteristic peaks at 200 cm^−1^, 400 cm^−1^, and 600 cm^−1^, which is consistent with literature reports [43,44]. Judging from the spectra of the composite films, there are characteristic peaks of both carbon and Ti_3_C_2_Tx. This indicates that Ti_3_C_2_T_x_/graphene has been composited with the CNT array. The XPS results for the composite film further confirm the successful incorporation of Ti_3_C_2_T_x_ and graphene nanosheets into the CNT array (Figure S3).

The conductivity of the initial CNT arrays and the composite films, with varying structural parameters, has been systematically analyzed (Figure 2b,c). Here, three different thicknesses of CNT arrays are grown, including 150, 250, and 350 μm. An area ratio is used to describe the proportion of CNT arrays in composite films. Specifically, the area ratio refers to the fraction of the area covered by the CNT array compared to the total area of the film. For CNT arrays with different structures, three samples with different area ratios are synthesized, namely 64%, 75%, and 84%. An area ratio that is too high or too low will make it difficult to transfer the composite film. The conductivity of the initial CNT array is relatively low because the CNTs are arranged in a directional manner, which results in weak lateral bonding between the tubes (Figure 2b). However, the introduction of Ti_3_C_2_T_x_/graphene nanosheets into the grids of the CNT array has significantly enhanced the conductivity of the composite film. Generally, a higher area ratio of the CNT array corresponds to increased conductivity. Similarly, an increase in the array thickness also leads to an elevation in conductivity. The conductivity of the CNT arrays with diamond patterns and triangular patterns are presented in Figure S4.

**Figure 3:**
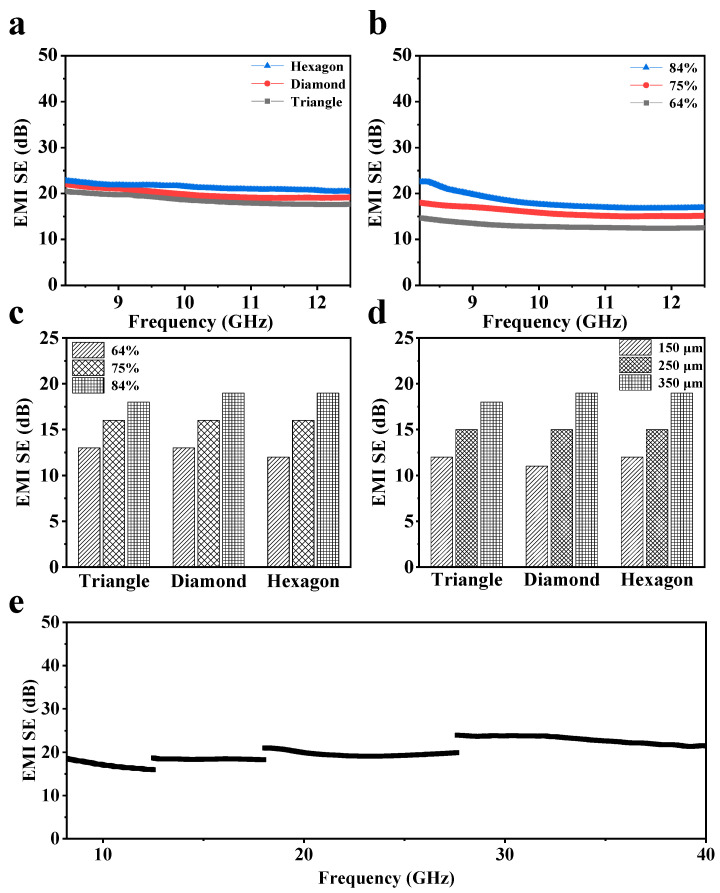
EMI SE of the initial CNT arrays. (**a**) EMI SE of the CNT array with different patterns at the same thickness (350 μm) and area ratio (84%). (**b**) EMI SE of the diamond-patterned CNT array with different area ratios under the same thickness (350 μm). (**c**) EMI SE of the CNT array with different area ratios under the same thickness (350 μm). (**d**) EMI SE of the CNT array with different thicknesses under the same area ratio (84%). (**e**) EMI SE of the CNT array in the band of 8.2–40 GHz.

To investigate how the parameters of pattern structures impact the electromagnetic shielding characteristics of initial CNT films, the EMI SE within the X-band (8.2–12.4 GHz) is systematically analyzed. As shown in Figure 3a, CNT arrays with the same thickness (350 μm) and area ratio (84%) but different pattern structures exhibit similar EMI SE in the X-band. The CNT array with a hexagonal pattern has an EMI SE of approximately 18.9 dB, indicating that only about 1% of the incident electromagnetic waves can pass through the sample. However, the area ratio of CNT arrays has a significant impact on the electromagnetic shielding performance (Figure 3b). For the CNT arrays with a diamond pattern and a thickness of 350 μm, the sample with an area ratio of 84% demonstrates the best average EMI SE (18.1 dB), followed by the array with a 75% area ratio at 15.9 dB, and the one with a 64% area ratio at 13.0 dB. Figure 3c,d have compared the EMI SE of CNT arrays with different patterns while maintaining a fixed film thickness of 350 μm or a constant area ratio of 84%, respectively. The results indicate that CNT arrays with different pattern structures exhibit similar trends in their influence on EMI SE. The EMI SE of the continuous CNT array film of the same thickness is presented in Figure S5. Compared with the patterned CNT array, the continuous array film demonstrates better EMI SE, primarily due to its higher area ratio. The initial hexagonal-patterned CNT array film, which has an area ratio of 84% and a thickness of 350 μm, also exhibits good shielding performance in Ku, K, and Ka bands (Figure 3e). The average SE reaches 19.5 dB within the frequency range of 8.2–40 GHz.

**Figure 4:**
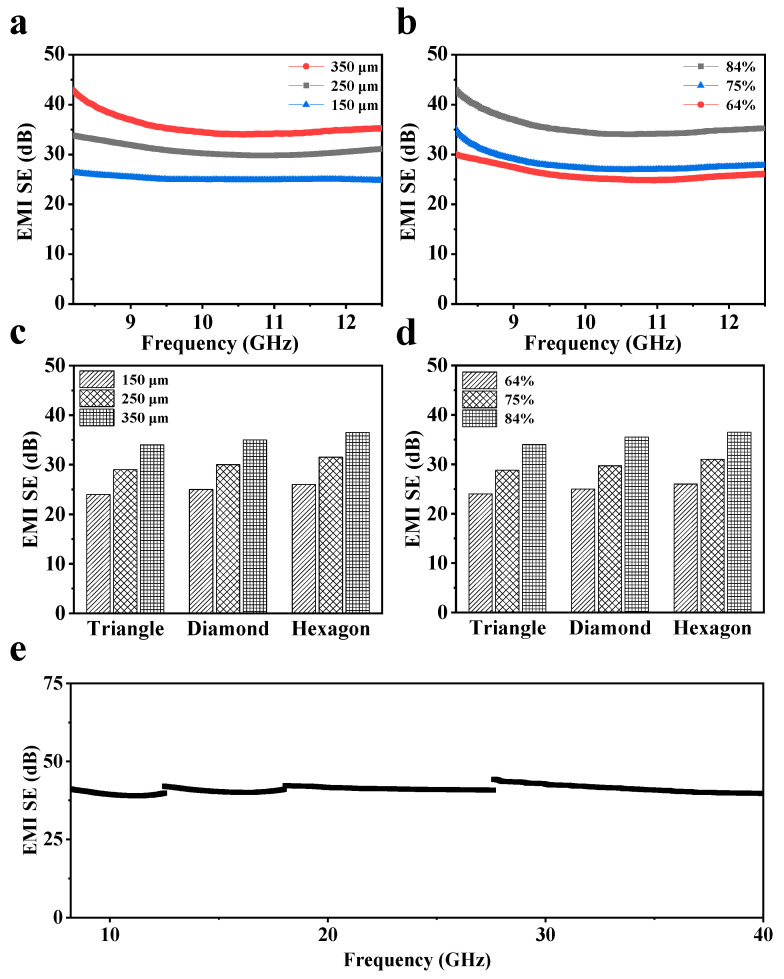
EMI SE of the composite films. (**a**) EMI SE of the composite films with a hexagonal pattern and the same area ratio (84%). (**b**) EMI SE of the composite films with a hexagonal pattern and the same thickness (350 μm). (**c**) EMI SE of the composite films with different thicknesses under the same area ratio (84%). (**d**) EMI SE of the composite films with different area ratios under the same thickness (350 μm). (**e**) EMI SE of the composite film in the band of 8.2–40 GHz.

Both Ti_3_C_2_T_x_ and graphene possess high electrical conductivity. To endow the sample with better shielding performance, the same CNT arrays are filled with three different slurries: a Ti_3_C_2_T_x_/graphene mixed slurry, a Ti_3_C_2_T_x_-only slurry, and a graphene-only slurry. The EMI SE results of the composite films obtained by scraping the above-mentioned slurries are shown in Figure S6 and Table S1. The EMI SE results reveal that the film coated with the Ti_3_C_2_T_x_/graphene provides the best performance. In contrast, the graphene-only composite film demonstrates lower electrical conductivity, primarily due to the agglomeration and stacking of nanosheets in the water-based graphene mixture. When only Ti_3_C_2_T_x_ is used for the scraping coating, the Ti_3_C_2_T_x_ filling the “cells” of the CNT array tends to stick to the substrate, making it difficult to peel off and resulting in an incomplete composite film. In contrast, the mixed slurry can effectively carry Ti_3_C_2_T_x_ into the “cells”, significantly enhancing the electrical conductivity and SE of the composite film. Figure 4a illustrates the impact of the array thickness on the EMI SE. As the thickness increases, the content of Ti_3_C_2_T_x_/graphene filled in the “cells” also increases. Correspondingly, the electrical conductivity and shielding performance of the composite film are enhanced. The average EMI SE of the composite film with a thickness of 350 μm reaches 36.5 dB, which is considerably higher than that of the CNT array without filling with Ti_3_C_2_T_x_/graphene (19.3 dB). For the composite films with a hexagonal pattern, as the array area ratio increases from 64% to 84%, the EMI SE rises from 26.4 dB to 36.5 dB (Figure 4b). Since a larger area ratio of the CNT array is more conducive to forming a stable conductive network structure, increasing the area ratio of the CNT array shows a positive impact on the EMI SE of composite films, similar to the initial pattern-structured CNT array (Figure 3c). In addition, the reflection proportion (*R*) of the film to incident electromagnetic waves exceeds 90% (Figure S7), which indicates that reflection is the main shielding mechanism. Figure 4c,d show the influence of array thickness or area ratio on SE, respectively. The composite films with different patterns demonstrate similar EMI SE values. Nevertheless, for all of these patterned samples, the EMI SE improves with the increase in thickness and area ratio of the CNT array. These results correlate with their electrical conductivities (Figure 2c and Figure S3b,d). The detailed effects of the array thickness and area ratio on the EMI SE of films with various patterns are detailed in Figures S8–S10, respectively. The comprehensive statistics and comparisons of the electrical conductivity and EMI SE of hexagon, diamond, and triangle patterns with different parameters are presented in Tables S2–S4, respectively. Figure 4e illustrates the EMI SE of the composite hexagonal-patterned film across the 8.2–40 GHz range, featuring an area ratio of 84% and a thickness of 350 μm. The EMI SE of the composite film across Ku, K, and Ka bands has also witnessed an outstanding enhancement, besides the X band. Overall, the average EMI SE is approximately 40.7 dB, representing an improvement of 103.6% compared to the original CNT array (19.5 dB).

**Figure 5:**
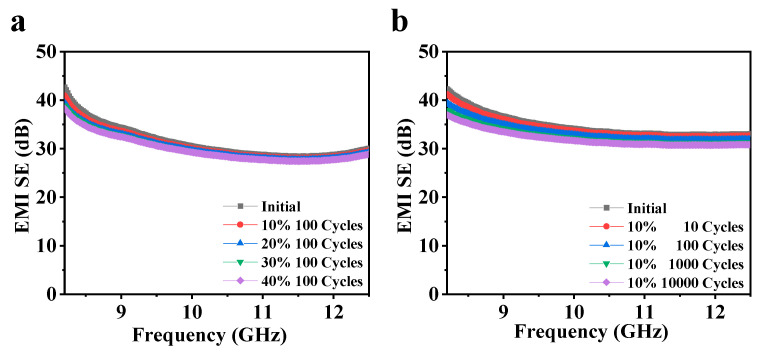
The stability of the EMI SE of the composite films. (**a**) EMI SE of the composite film (hexagonal pattern, 350 μm, and 84% area ratio) after 100 cycles of stretching under different tensile strains (0–40%). (**b**) EMI SE of the composite film (hexagonal pattern, 350 μm, and 84% area ratio) after 10,000 stretching cycles at the strain of 10%.

The stability and stretchability of the composite film have been systematically tested. Figure 5a presents the EMI SE of the composite film after 100 cycles of stretching at various tensile strains (10–40%). The composite film exhibits excellent stability, and even after 100 cycles of stretching at a strain of up to 40%, the EMI SE only decreases from 32.2 dB to 30.1 dB, representing a reduction of approximately 6.5%. The reason for the decrease is that during the stretching process, some of the conductive network of the composite film may by damaged, which affects its conductivity and electromagnetic shielding performance. Similarly, after 10,000 stretching cycles at a strain of 10%, the EMI SE of the composite film only declines from 34.8 dB to 32.5 dB, which is a reduction of 6.6% (Figure 5b). The composite film after being stretched can still achieve over 99.9% isolation from electromagnetic waves, and the EMI SE remains basically stable. The EMI SE of composite films with other patterns after varying stretching cycles is displayed in Figure S11 and Figure S12, respectively, which are similar to those of the film with a hexagon pattern. The combination of CNT with other materials can also achieve excellent EMI SE and microwave absorption performance [45,46,47]. In comparison, the CNT array/Ti_3_C_2_T_x_/graphene composite film in this work possesses better tensile resistance and more stable EMI SE within the 8–40 GHz range.

**Figure 6:**
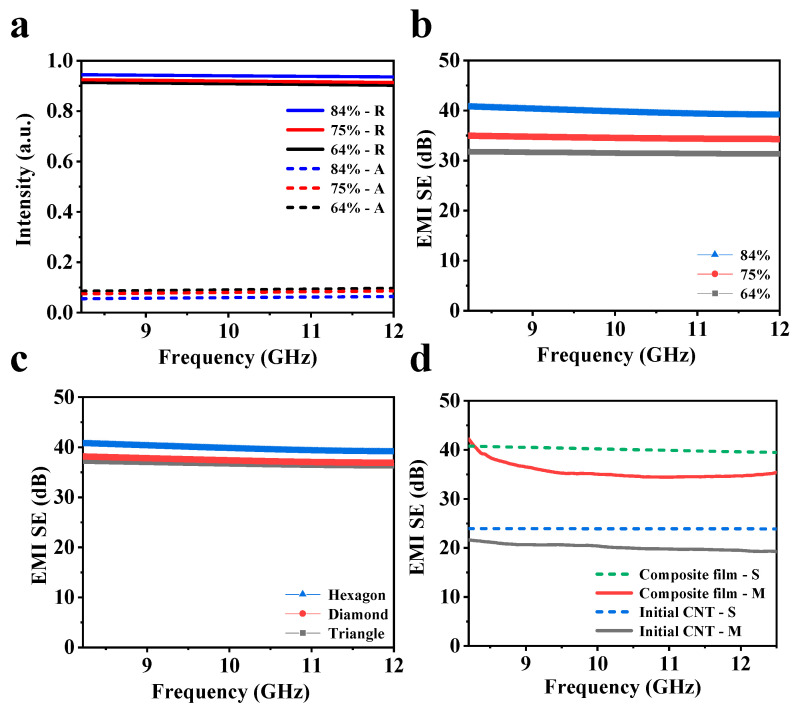
Simulation of EMI SE of composite films. (**a**) Reflection (R) and absorption(A) ratios of samples (hexagonal pattern, 350 μm) with different area ratios. (**b**) EMI SE of samples (hexagonal pattern, 350 μm) with different area ratios. (**c**) EMI SE of samples (350 μm, 84% area ratio) with different patterns. (**d**) Measured (M) and simulated (S) EMI SE of initial CNT array and composite films.

To investigate the shielding mechanism of the composite film, the interaction process between the composite film and electromagnetic waves is simulated using the software CST (CST Studio Suite 2022, v1.0.0). By modeling the complex frequency-dependent s-parameters (S11 and S12), the reflection (*R*), absorption (*A*), and transmission (*T*) ratio of the composite material can be realized, and computations can be performed using the shielding formula to obtain the EMI SE. During the simulation, based on the Floquet model [41], a tetrahedral mesh founded on the finite element method and a frequency-domain solver are employed for simulation. The periodic structures of the three patterns of CNT arrays are shown in Figure S13a–c, including hexagonal, diamond, and triangle patterns. The *A* and *R* ratios for the hexagonal films (with a thickness of 350 μm) at different area ratios are presented in Figure 6a. Additionally, by calculating the transmission ratio, the EMI SE of the composite films can also be achieved (Figure 6b). As the area ratio increases, the reflection ratio rises from 0.9 to 0.94, resulting in an increase in the EMI SE of the sample from 31.8 dB to 39.6 dB (Figure S13g–i). The *A*, *R*, and *EMI SE* of the films with other patterns are illustrated in Figure S13d–f, showing similarity to the hexagon-patterned one. This verifies that the shielding mechanism of the composite material is dominated by reflection. Under the same array thickness (350 μm) and area ratio (84%), the EMI SE of the films with hexagonal, diamond, and triangle patterns is 39.4 dB, 37.9 dB, and 37.2 dB, respectively. These results indicate that samples with a hexagonal pattern provide the best shielding, and therefore align with the experimental findings. The comparison between the simulation and the experimental results is exhibited in Figure 6d, showcasing the films before and after compounding with Ti_3_C_2_T_x_/graphene, respectively. The parameters of the simulated structure are in accordance with those of the prepared films (Figure S13a–c). In experimental tests, after compositing with Ti_3_C_2_T_x_/graphene, the EMI SE of CNT arrays increases by 67.4% (from 21.8 dB to 36.5 dB). The simulation calculations support this conclusion, indicating an increase of 69.6% in EMI SE, from 23.7 dB to 40.2 dB after the composite is applied. These findings further confirm that surface reflection is the primary shielding mechanism for the composite films.

## 4. Conclusions

In summary, we report a simple scraping and coating process to construct stretchable and patterned CNT array/Ti_3_C_2_T_x_/graphene composite films with high EMI shielding performance. The results indicated that after compounding with Ti_3_C_2_T_x_/graphene, the average electromagnetic shielding effectiveness of the patterned CNT array has been significantly optimized and improved (increased from 21.3 dB to 36.5 dB). Factors such as the array thickness, area ratio, and pattern morphology show various impacts on the electrical conductivity and shielding performance of the composite samples. The results of the tensile fatigue tests indicate that the composite films process extremely good tensile resistance and stability. In addition, the CST simulation software (CST Studio Suite 2022, v1.0.0) is used to design the composite samples, and it is verified that reflection is the main shielding mechanism. The patterned CNT array/Ti_3_C_2_T_x_/graphene composite films exhibit application prospects in flexible shielding.

## Data Availability

The original contributions presented in this study are included in the article and Supplementary Material. Further inquiries can be directed to the corresponding author.

## Supplementary Materials

The following supporting information can be downloaded at www.mdpi.com/article/10.3390/nano15050391/s1: Figure S1: SEM images of cross-sections of CNT arrays with different thicknesses. Figure S2: High magnification SEM image of composite films. Figure S3: XPS spectra of the composite film. Figure S4: Electrical conductivity of the initial CNT arrays and composite films. Figure S5: EMI SE of continuous CNT arrays (without any “cells”) with different thicknesses. Figure S6: The EMI SE of composite films filled with Ti_3_C_2_T_x_/graphene, Ti_3_C_2_T_x_, and graphene, respectively. Figure S7: Absorption (*SE_A_*), reflection (*SE_R_*), and transmission (*SE_T_*) shielding effectiveness of the composite films with hexagonal patterns (a thickness of 350 μm). Figure S8: Influence of array thicknesses or area ratios on EMI SE of the composite films with hexagon pattern. Figure S9: Influence of array thicknesses or area ratios on EMI SE of the composite films with diamond pattern. Figure S10: Influence of array thicknesses or area ratios on EMI SE of the composite films with triangle pattern. Figure S11: The stability of the EMI SE of the composite films. Figure S12: The stability of the EMI SE of the composite films. Figure S13: Simulation of EMI SE of composite films. Table S1: Electromagnetic shielding effectiveness of different films. Tables S2–S4. Electrical conductivity and average EMI SE of composite film with hexagon, diamond, and triangle patterns, respectively.
