# Supplementary material for: Stretchable, Patterned Carbon Nanotube Array Enhanced by Ti3C2Tx/Graphene for Electromagnetic Interference Shielding
Source: Nanomaterials (Basel). 2025 Mar 3;15(5):391. doi: 10.3390/nano15050391 (PMC11902016; doi:10.3390/nano15050391)
Supplement: Supplementary file 1 [file nanomaterials-15-00391-s001.zip › nanomaterials-3474177-supplementary.pdf]

Supporting Information

Stretchable, Patterned Carbon Nanotube Array Enhanced by $Ti_3C_2T_x$/graphene for Electromagnetic Interference Shielding

Baohua Li[1], Xuebin Liu[1], Jiyong Feng[1], Yunfan Wang[1], Junhua Huang[1], Zhengwei Fu[2], Zhiping Zeng[3], Jianghui Zheng[*2], and Xuchun Gui [*1]

[1]*State Key Laboratory of Optoelectronic Materials and Technologies, School of Electronics and Information Technology, Sun Yat-sen University, Guangzhou, 510275, China*

[2]*Beijing Zhenxing Institute of Metrology and Measurement, Beijing, 100074, China*

[3]*School of Materials Science and Engineering, Sun Yat-sen University, Guangzhou, 510275, China*

*Correspondence: *guixch@mail.sysu.edu.cn; 18310265803@163.com*

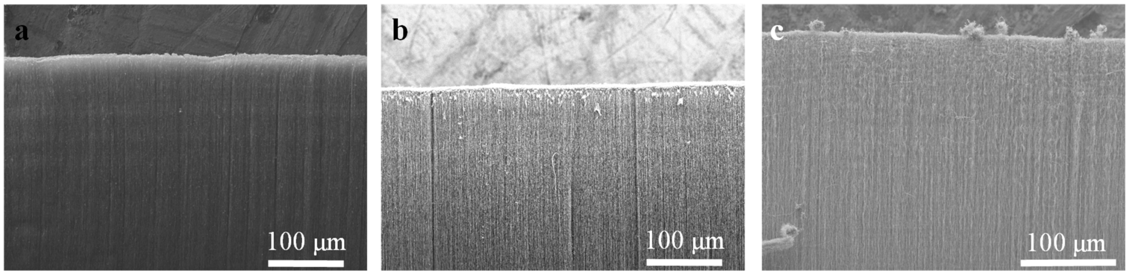

Figure S1. SEM images of cross-sections of CNT arrays with different thicknesses.

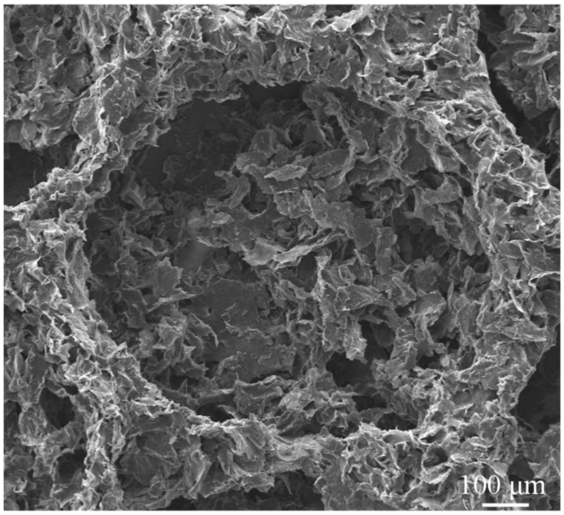

Figure S2 High magnification SEM image of composite films with the periodic hexagonal patterns.

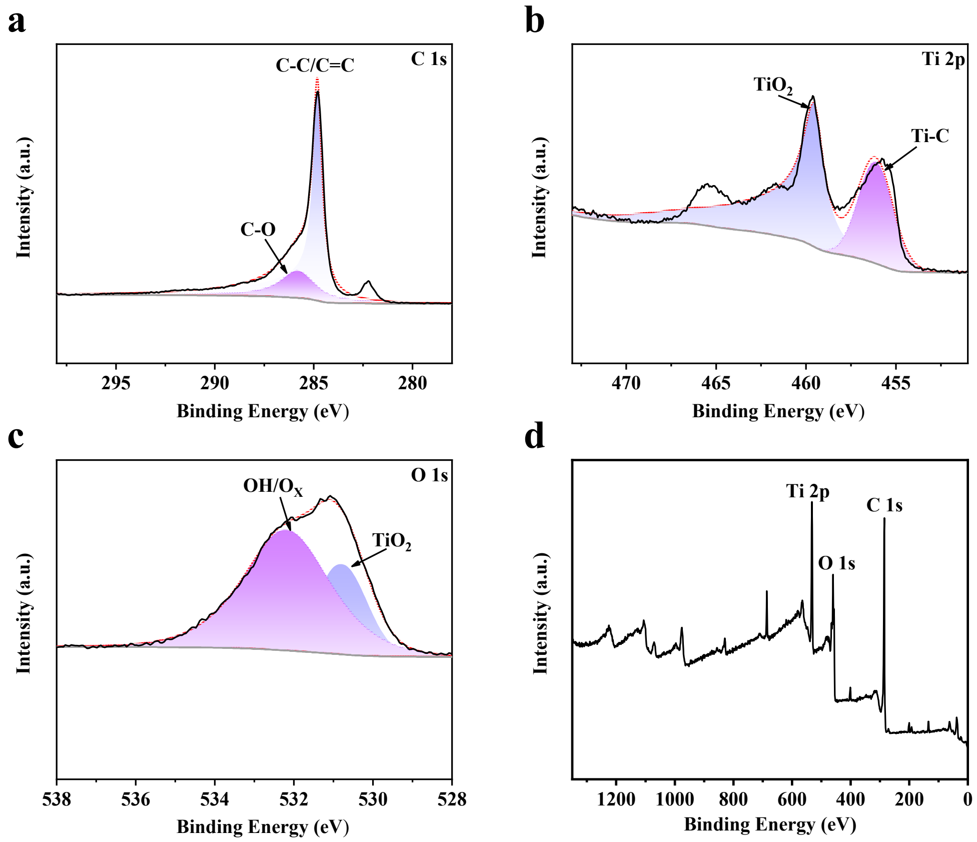

Figure S3. XPS spectra of the composite film. (**a**) High-resolution XPS spectrum of C 1s. (**b**) High-resolution XPS spectrum of Ti 2p. (**c**) High-resolution XPS spectrum of O 1s. (**d**) XPS full spectrum of the sample.

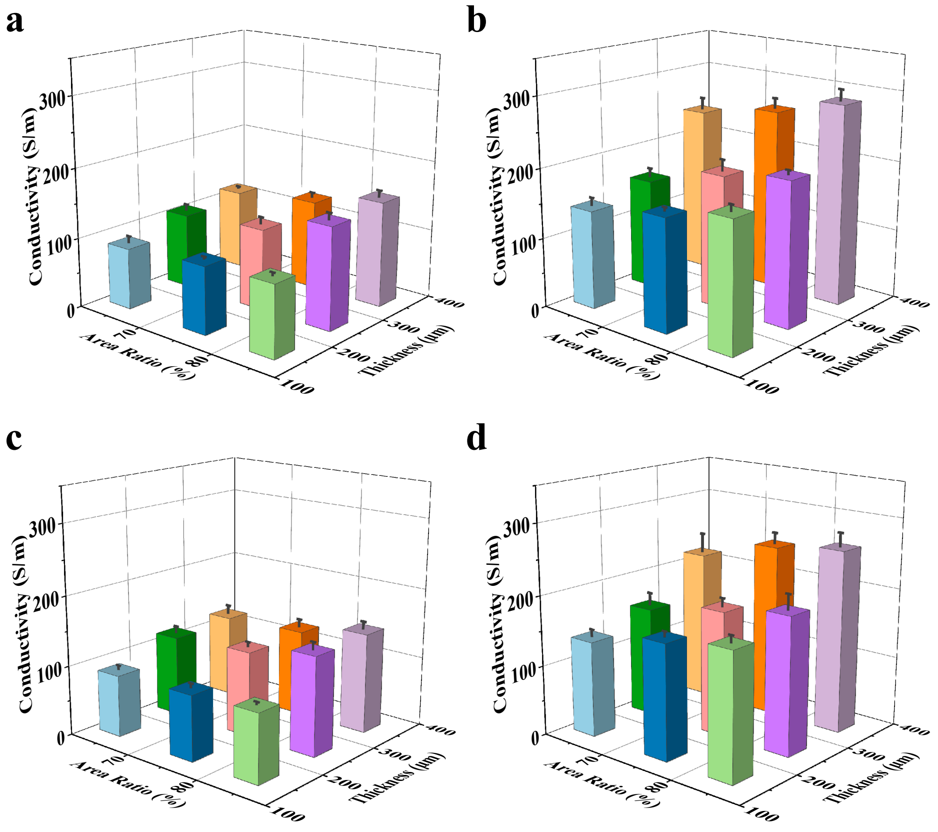

Figure S4. Electrical conductivity of the initial CNT arrays and composite films. (**a**) The conductivity of the initial CNT arrays with the diamond pattern at different thicknesses and area ratios. (**b**) The conductivity of the composite films with the diamond pattern at different thicknesses and area ratios. (**c**) The conductivity of the initial CNT arrays with the triangle pattern at different thicknesses and area ratios. (**d**) The conductivity of the composite films with the triangle pattern at different thicknesses and area ratios.

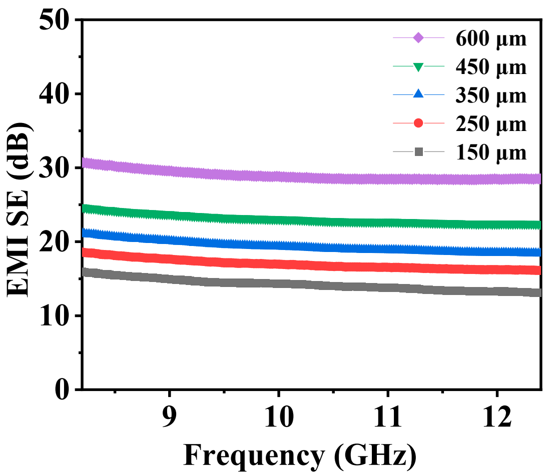

Figure S5. EMI SE of continuous CNT arrays (without any “cells”) with different thicknesses.

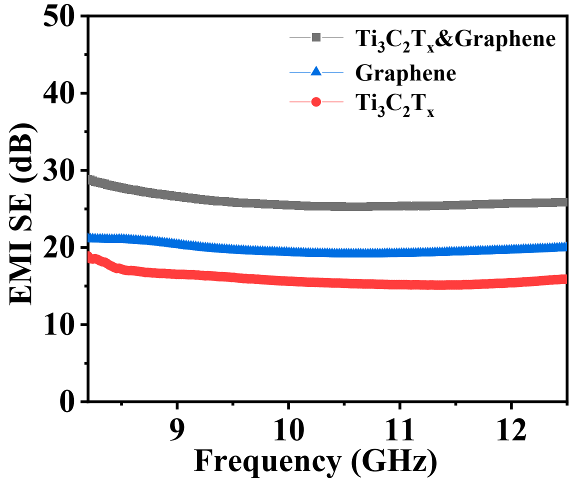

Figure S6. The EMI SE of composite films filled with $Ti_3C_2T_x$/graphene, $Ti_3C_2T_x$, and graphene, respectively.

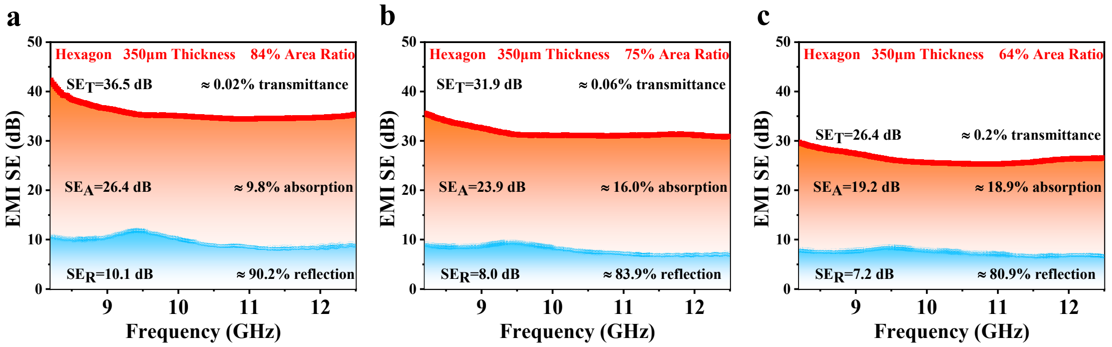

Figure S7. Absorption (*SE_A*), reflection (*SE_R*), and transmission (*SE_T*) shielding effectiveness of the composite films under different area ratios (hexagonal pattern, 350 μm thickness). (**a**) 84% area ratio. (**b**) 75% area ratio. (**c**) 64% area ratio.

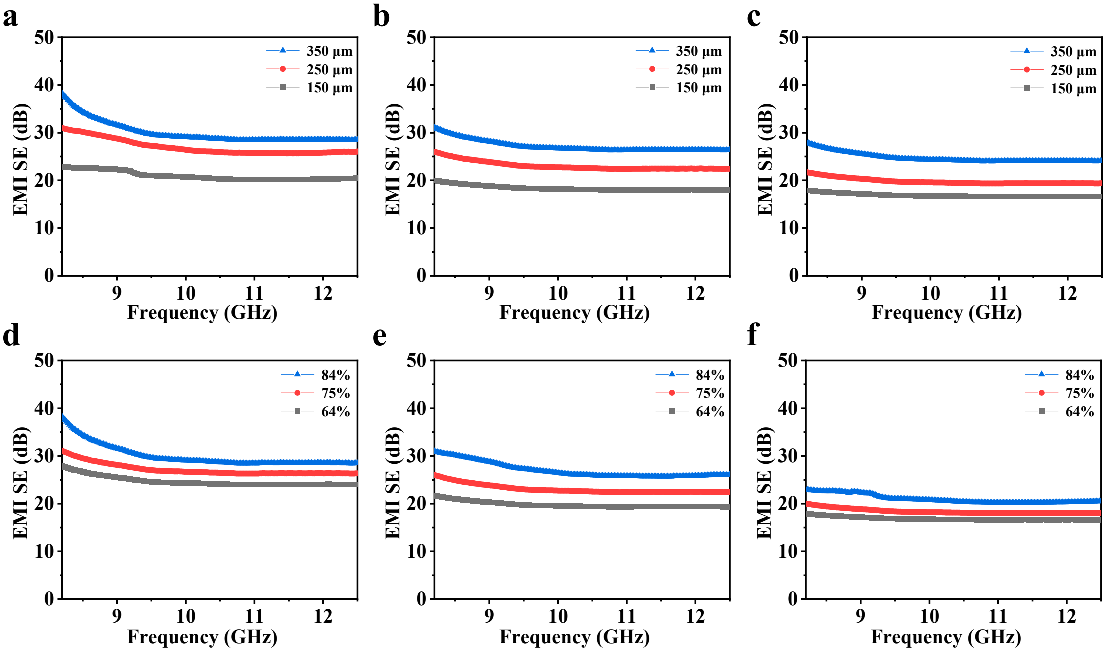

Figure S8. Influence of array thicknesses or area ratios on EMI SE of the composite films with hexagon patterns. (**a**) 84% area ratio. (**b**) 75% area ratio. (**c**) 64% area ratio. (**d**) 350 μm. (**e**) 250 μm. (**f**) 150 μm.

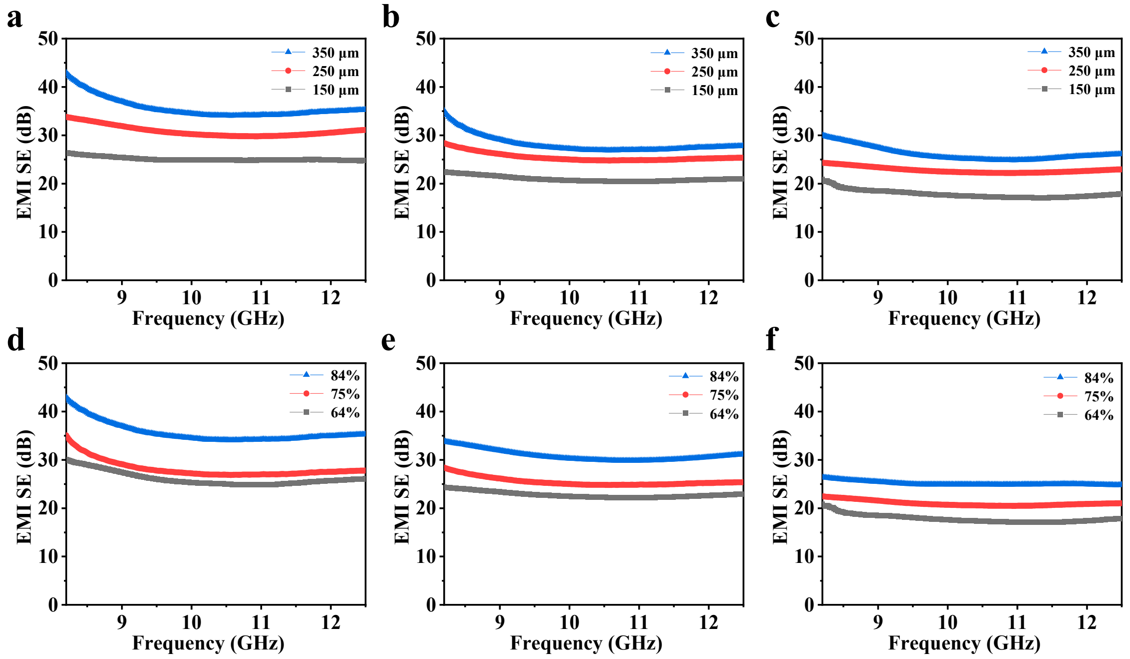

Figure S9. Influence of array thicknesses or area ratios on EMI SE of the composite films with diamond patterns. (**a**) 84% area ratio. (**b**) 75% area ratio. (**c**) 64% area ratio. (**d**) 350 μm. (**e**) 250 μm. (**f**) 150 μm.

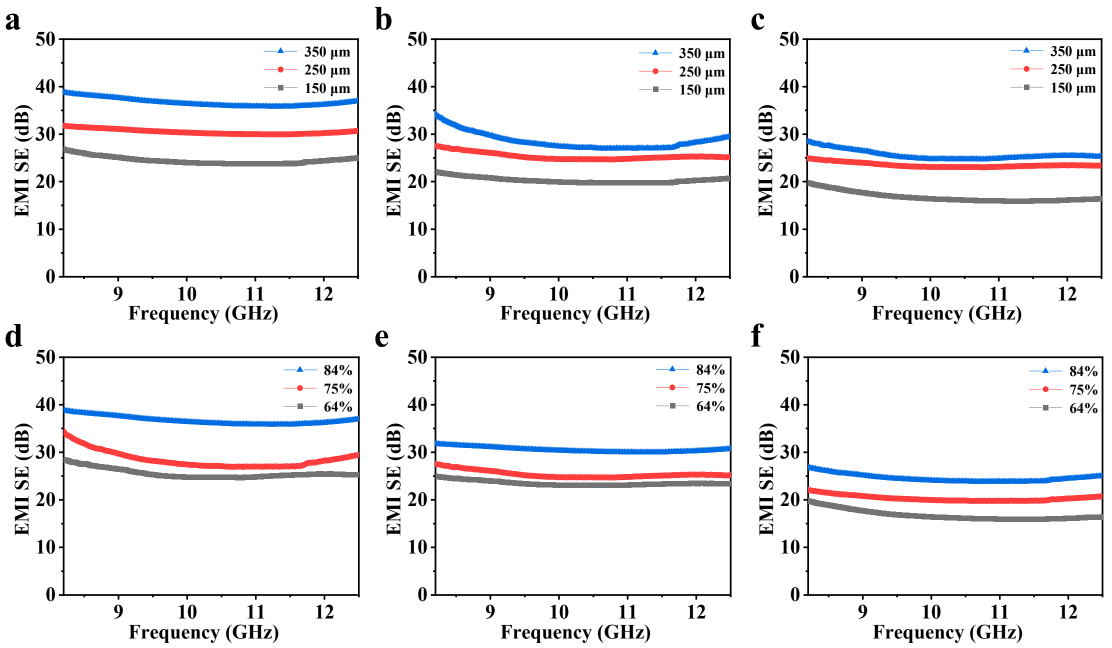

Figure S10. Influence of array thicknesses or area ratios on EMI SE of the composite films with triangle patterns. (**a**) 84% area ratio. (**b**) 75% area ratio. (**c**) 64% area ratio. (**d**) 350 μm. (**e**) 250 μm. (**f**) 150 μm.

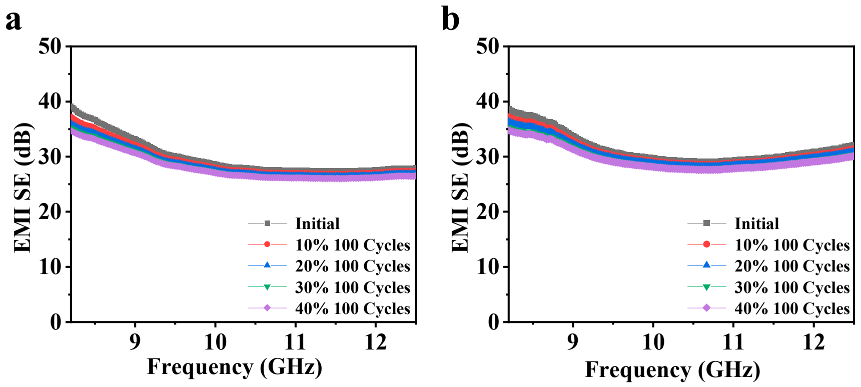

Figure S11. The stability of the EMI SE of the composite films. (**a**) EMI SE of the composite film (diamond pattern, 350 μm, and 84% area ratio) after 100 cycles of stretching under different tensile strains (10-40%). (**b**) EMI SE of the composite film (triangle pattern, 350 μm, and 84% area ratio) after 100 cycles of stretching under different tensile strains (10-40%).

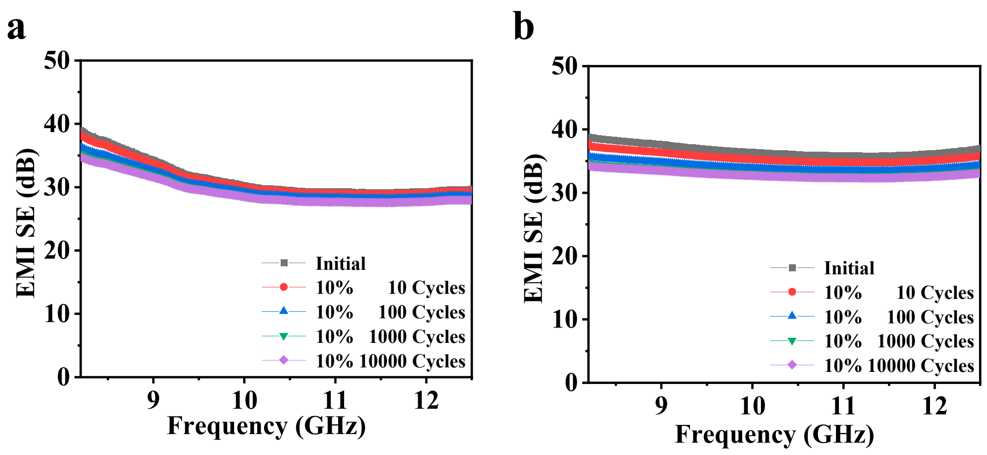

Figure S12. The stability of the EMI SE of the composite films. (**a**) EMI SE of the composite film (diamond pattern, 350 μm, and 84% area ratio) after 10,000 stretching cycles at the tensile strain of 10%. (**b**) EMI SE of the composite film (triangle pattern, 350 μm, and 84% area ratio) after 10,000 stretching cycles at the tensile strain of 10%.

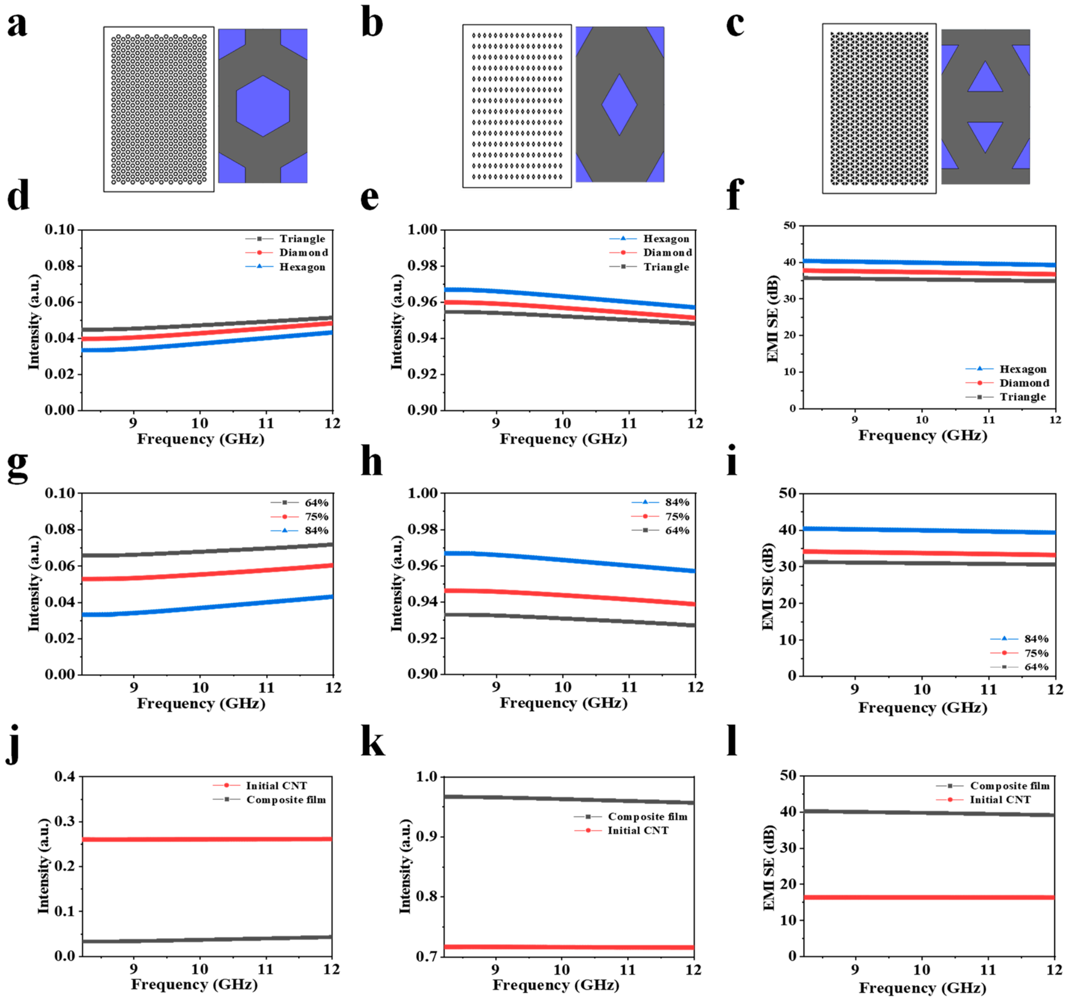

Figure S13. Simulation of EMI SE of composite films. (**a-c**) Simulation designs with different patterns. (**d**) Absorption(A) ratios of composite films (350 μm, 84% area ratio) with different patterns. (**e**) Reflection(R) ratios of composite films (350 μm, 84% area ratio) with different patterns. (**f**) EMI SE of composite films (350 μm, 84% area ratio) with different patterns. (**g**) Absorption(A) ratios of composite films (hexagonal pattern, 350 μm) with different area ratios. (**h**) Reflection (R) ratios of composite films (hexagonal pattern, 350 μm) with different area ratios. (**i**) EMI SE of composite films (hexagonal pattern, 350 μm) with different area ratios. (**j**) Absorption(A) ratios of the initial CNT array and the composite film (350 μm, 84% area ratio). (**k**) Reflection(R) ratios of the initial CNT array and the composite

film (350 μm, 84% area ratio). (**l**) EMI SE of the initial CNT array and the composite film (350 μm, 84% area ratio).

Table S1. Electromagnetic shielding effectiveness of different film.

EMI SE (dB) / Films / Samples	Initial CNT arrays	Composite films filled with $Ti_3C_2T_x$	Composite films filled with graphene	Composite films filled with $Ti_3C_2T_x$/graphene
With Diamond patterns<br>Thickness of 250 μm<br>Area Ratio of 75%	**14.2**	**15.9**	**19.7**	**26.0**
With Triangle patterns<br>Thickness of 350 μm<br>Area Ratio of 75%	**16.4**	**17.5**	**20.2**	**27.8**
With Hexagon patterns<br>Thickness of 350 μm<br>Area Ratio of 64%	**21.5**	**24.1**	**25.7**	**36.5**

Table S2. Electrical conductivity and average EMI SE of composite film (filled with $Ti_3C_2T_x$/graphene) with hexagon patterns at different area ratio and thickness.

**Area Ratio (%)**	**Thickness (μm)**	**Electricity of initial CNT arrays (S/m)**	**Electricity of the composite film (S/m)**	**EMI SE of initial CNT arrays (dB)**	**EMI SE of the composite film (dB)**
64	150	91.2	147.3	8.1	18.5
75	150	99.5	169.8	10.4	22.8
84	150	100.4	185.7	12.2	26.1
64	250	113.5	168.3	10.1	22.4
75	250	127.4	196.2	12.3	27.2
84	250	141.5	216.3	15.5	33.9
64	350	128.7	250.8	12.7	26.1
75	350	147.4	272.9	16.4	31.1
84	350	158.4	312.3	19.2	36.5

Table S3. Electrical conductivity and average EMI SE of composite film (filled with $Ti_3C_2T_x$/graphene) with diamond patterns at different area ratio and thickness

**Area Ratio (%)**	**Thickness (μm)**	**Electricity of initial CNT arrays (S/m)**	**Electricity of the composite film (S/m)**	**EMI SE of initial CNT arrays (dB)**	**EMI SE of the composite film (dB)**
64	150	87.4	141.7	8.2	17.5
75	150	96.5	161.3	10.5	20.7
84	150	101.4	183.7	12.9	25.3
64	250	107.7	158.8	11	22.9
75	250	116.9	191.1	12.5	25.7
84	250	146.5	206.7	15.5	31.5
64	350	115.7	240.8	13.8	25.2
75	350	128.5	260.7	16.6	29.4
84	350	151.8	286.6	19.7	35.2

Table S4. Electrical conductivity and average EMI SE of composite film (filled with $Ti_3C_2T_x$/graphene) with triangle patterns at different area ratio and thickness

**Area Ratio (%)**	**Thickness (μm)**	**Electricity of initial CNT arrays (S/m)**	**Electricity of the composite film (S/m)**	**EMI SE of initial CNT arrays (dB)**	**EMI SE of the composite film (dB)**
64	150	88.8	136.4	8.1	17.8
75	150	93.7	163.8	10.4	19.4
84	150	97.8	178.6	12.8	24.6
64	250	115.4	157.5	11.3	20.5
75	250	122.2	179.8	13.5	24.8
84	250	141.7	195.6	15.7	29.3
64	350	119.4	216.8	13.1	25.8
75	350	126.4	244.5	16.6	28.7
84	350	143.7	264.3	18.9	35.0
